# Spectroscopic Studies of Quinobenzothiazine Derivative in Terms of the In Vitro Interaction with Selected Human Plasma Proteins. Part 1

**DOI:** 10.3390/molecules26164776

**Published:** 2021-08-06

**Authors:** Aleksandra Owczarzy, Andrzej Zięba, Jadwiga Pożycka, Karolina Kulig, Wojciech Rogóż, Agnieszka Szkudlarek, Małgorzata Maciążek-Jurczyk

**Affiliations:** 1 Department of Physical Pharmacy, Faculty of Pharmaceutical Sciences in Sosnowiec, Medical University of Silesia in Katowice, 40-055 Katowice, Poland; aowczarzy@sum.edu.pl (A.O.); jpozycka@sum.edu.pl (J.P.); kkulig@sum.edu.pl (K.K.); wrogoz@sum.edu.pl (W.R.); aszkudlarek@sum.edu.pl (A.S.); 2 Department of Organic Chemistry, Faculty of Pharmaceutical Sciences in Sosnowiec, Medical University of Silesia in Katowice, 40-055 Katowice, Poland; zieba@sum.edu.pl

**Keywords:** plasma proteins, 5-alkyl-12(H)-quino[3,4-b][1,4]benzothiazinium derivative, spectroscopy

## Abstract

Plasma proteins play a fundamental role in living organisms. They participate in the transport of endogenous and exogenous substances, especially drugs. 5-alkyl-12(H)-quino[3,4-b][1,4]benzothiazinium salts, have been synthesized as potential anticancer substances used for cancer treatment. Most anticancer substances generate a toxic effect on the human body. In order to check the toxicity and therapeutic dosage of these chemicals, the study of ligand binding to plasma proteins is very relevant. The present work presents the first comparative analysis of the binding of one of the 5-alkyl-12(H)-quino[3,4-b][1,4]benzothiazinium derivatives (Salt1) with human serum albumin (HSA), α-1-acid glycoprotein (AGP) and human gamma globulin (HGG), assessed using fluorescence, UV-Vis and CD spectroscopy. In order to mimic in vivo ligand–protein binding, control normal serum (CNS) was used. Based on the obtained data, the Salt1 binding sites in the tertiary structure of all plasma proteins and control normal serum were identified. Both the association constants (K_a_) and the number of binding site classes (*n*) were calculated using the Klotz method. The strongest complex formed was Salt1–AGP_complex_ (K_a_ = 7.35·10^4^ and 7.86·10^4^ mol·L^−1^ at excitation wavelengths λ_ex_ of 275 and 295 nm, respectively). Lower values were obtained for Salt1–HSA_complex_ (K_a_ = 2.45·10^4^ and 2.71·10^4^ mol·L^−1^) and Salt1–HGG_complex_ (K_a_ = 1.41·10^4^ and 1.33·10^4^ mol·L^−1^) at excitation wavelengths λ_ex_ of 275 and 295 nm, respectively, which is a positive phenomenon and contributes to the prolonged action of the drug. Salt1 probably binds to the HSA molecule in Sudlow sites I and II; for the remaining plasma proteins studied, only one binding site was observed. Moreover, using circular dichroism (CD), fluorescence and UV-Vis spectroscopy, no effect on the secondary and tertiary structures of proteins in the absence or presence of Salt1 has been demonstrated. Despite the fact that the conducted studies are basic, from the scientific point of view they are novel and encourage further in vitro and in vivo investigations. As a next part of the study (Part 2), the second new synthetized quinobenzothiazine derivative (Salt2) will be analyzed and published.

## 1. Introduction

Cancer diseases are an increasingly common cause of death. Due to these problems, the synthesis of new anticancer substances is a primary goal of organic chemists worldwide.

Plasma proteins constitute a very diverse group. Depending on the function performed, there are transporting proteins, proteins responsible for coagulation and immune processes and complex regulatory functions [1]. Human proteins such as human serum albumin (HSA), α-1-acid glycoprotein (AGP, orosomucoid), human gamma globulin (HGG) and lipoproteins have the ability to bind exogenous and endogenous substances. The most important transporting proteins are HSA and AGP, which play an important role in clinical pharmacokinetics (PK) and in pharmacodynamics (PD) studies [2].

Human serum albumin (HSA) is synthesized in the liver and represents 60% of the total plasma protein. The normal concentration of HSA in blood serum is 35–50 g·L^−1^. The ampholyte has the ability to bind cations and anions, so it can bind ligands with a different chemical nature [3,4]. The binding capacity of HSA alters ligands’ pharmacokinetic and pharmacodynamics properties. As a drug transporter, it improves the targeting of drugs and reduces the level of their side effects. It is worth noting that HSA binds ligands in the bloodstream at about 60%, and only the free fraction of drugs (unbound with protein) causes a pharmacological effect [5]. HSA is a three-dimensional, heart-shaped polypeptide. The molecular weight of HSA is 66.5 kDa, and the approximate dimensions are 80 Å 80 Å·30 Å. HSA contains 585 amino acids and has only one tryptophanyl residue at position 214 (Trp-214). The albumin structure consists of three α-helical domains, I–III, and each of them is divided into subdomains A and B [6]. In the albumin molecule, there are two sites with a characteristic structure and high affinity for drug binding called Sudlow sites I and II located in hydrophobic cavities in subdomains IIA and IIIA [7,8,9,10,11,12,13,14,15,16,17,18,19,20,21,22,23].

α-1-Acid glycoprotein (AGP, orosomucoid) is synthesized in the liver and is secreted into the circulation by hepatocytes. AGP belongs to the acute phase proteins. It is a carrier protein that is involved in the binding of alkaline medicinal substances. In response to stress, it modulates the immune system [24,25,26,27]. Protein concentration varies depending on the body state. Under physiological conditions, the concentration is in the range of 0.2–1 g·L^−1^, and the concentration increases two to three times in inflammation. Although AGP is present in the plasma in a small amount and its concentration varies significantly depending on the pathological process in the body, it plays an important role in the binding and transport of drugs. The free fraction of the drug depends on the AGP concentration in the plasma [25,28,29,30,31]. AGP molecular weight ranges from 41 to 43 kDa [13,26]. This molecule is a “highly acidic protein” and has a very low isoelectric point (pI 2.3–3.2) as a result of the presence of sialic acid, which constitutes 12% of the carbohydrate moiety [27,28]. The AGP molecule is composed of an 8-strand β-barrel surrounded by an α-helix. Eight β-strands form four loops (A/B, first loop; C/D, second loop; E/F, third loop; G/H, fourth loop), which make the entrance to the ligand pocket from the open end of the β-barrels. In the structure of α-1-acid glycoprotein, there are three tryptophanyl residues: Trp-25, found deep inside the β-barrel;Trp-122, found near the entrance to the drug-binding pocket and partially exposed to the water environment; and Trp-166, located on the surface of the molecule and completely exposed to the external environment [13,14]. Due to its physicochemical properties, AGP mainly binds neutral and alkaline drugs [32].

Gamma globulins (HGG) are other important components of plasma proteins. Their normal concentration ranges from 0.5 to 1.6 g·L^−1^ [1]. They function as part of the immune system but are also able to bind various metabolites and organic compounds, including drugs and appropriate antigens [33]. The binding of drugs to HGG plays an important role in monitoring therapy because it affects the concentration of the free drug fraction and thus its effectiveness and toxicity [34]. HGG is the most heterogeneous globulin with a molecular weight of approximately 150,000 Da. The HGG molecule is made up of four polypeptide chains, two light chains (L) and two heavy chains (H) which are connected by disulfide bonds and noncovalent interactions [19]. In the structures of chains, three areas can be distinguished: variable (V), located in the N-terminal segment, encoded by many genes of free amino acids; joining (J); and constant (C), terminated by a free carboxyl group, identical for all immunoglobulins. Between V and J regions, a diversity (D) region is present [1,15,16,17,18]. The human gamma globulin molecule consists of 49 tyrosyl and 20 tryptophanyl residues, which are probably involved in the ligand-binding processes [2,10].

The binding affinity of drugs to HSA and other plasma proteins is an important factor to consider during the design and development of new drugs [35,36,37,38,39,40]. The reversible, specific and nonspecific model of drugs binding to proteins is determined by drug physicochemical properties, pH and temperature [41]. These factors affect the time and the potency of the drug [42].

According to the World Health Organization (WHO), civilization diseases, especially cancer diseases, are the most common cause of death in the world. Since anticancer therapy has many side effects and an unsatisfactory effect, synthesis and study of new substances with anticancer potential are very important [43].

**Scheme 1:**
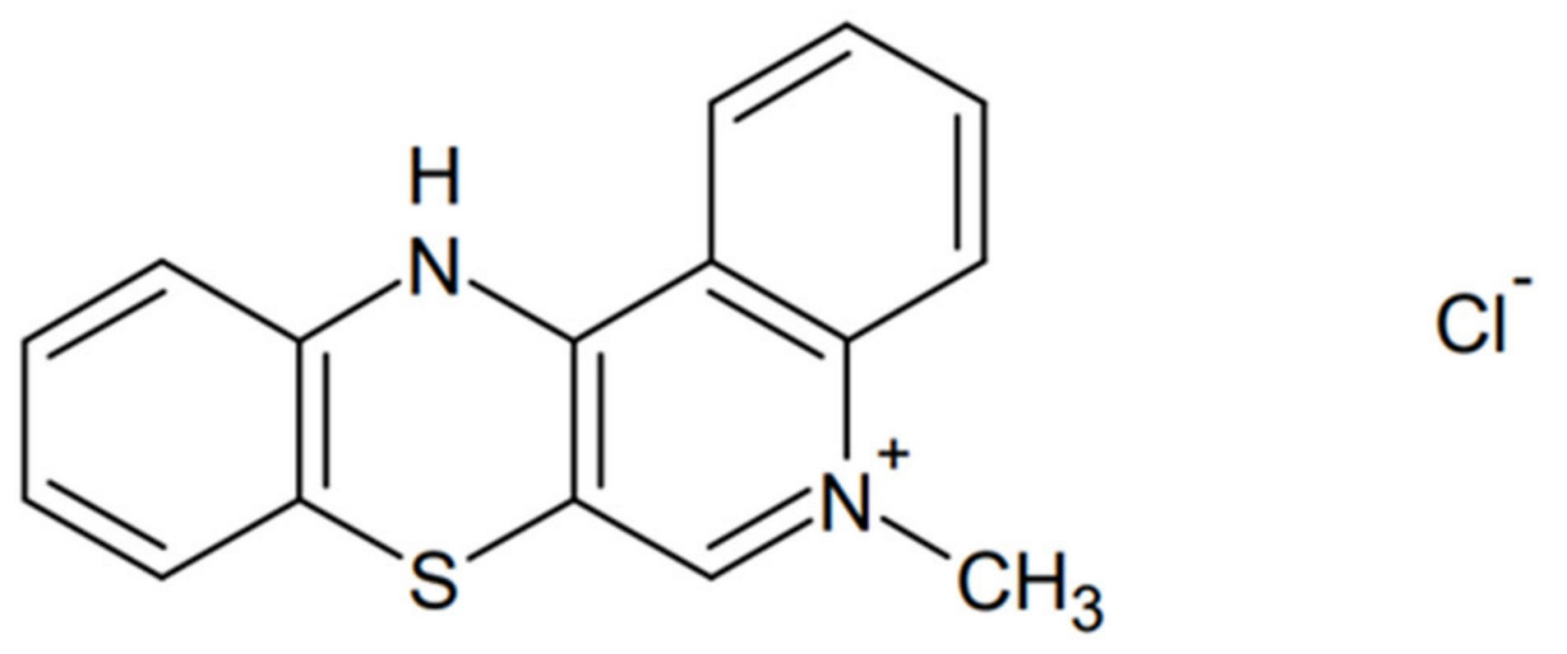
5-methyl-12(H)-chino[3,4-b][1,4]benzothiazinium chloride (Salt1) [44].

Among compounds that are being tested for anticancer activity are 5-alkyl-12(H)-quino[3,4-b][1,4]benzothiazinium derivatives, and one of them is 5-methyl-12(H)-chino[3,4-b][1,4]benzothiazinium chloride—Salt1 (Scheme 1).

5-Alkyl-12(H)-quino[3,4-b][1,4]benzothiazinium derivatives (Salt1 and Salt2) have been obtained in thioquinantrenium salt reactions with the corresponding arylamines. The antiproliferative activity of 5-alkyl-12(H)-quino[3,4-b][1,4]benzothiazinium salts was tested in vitro against the HCT 116 and LLC tumor cell lines using doxorubicin, which is currently one of the most important drugs in chemotherapy, as the reference compound. Antiproliferative activity reached an IC50 value in the range of 2.3–19.6 µg·mL^−1^. Dependence between the structure of the tested compounds and their antiproliferative activity has been observed. On the basis of the QSAR analysis, a correlation between the activity of the tested compounds and their lipophilic parameters, determined by reverse-phase thin layer chromatography and computational methods, has been determined [44,45,46].

Until now, no studies were examined quinobenzothiazine derivatives and proteins interaction. Thus, the aim of the current study was to compare the binding of 5-alkyl-12(H)-quino[3,4-b][1,4]benzothiazinium derivative 5-methyl-12(H)-chino[3,4-b][1,4]benzothiazinium chloride (Salt1) to different human plasma proteins (human serum albumin, α-1-acid glycoprotein and gamma globulin) and control normal serum—a protein mixture imitating the human serum used in the quality control of medical laboratories.

## 2. Results and Discussion

### 2.1. Salt1–HSA, Salt1–AGP, Salt1–HGG and Salt1–CNS Interaction

Based on the emission fluorescence spectra (data not shown) and fluorescence quenching curves (data not shown) of human serum albumin (HSA), α-1-acid glycoprotein (AGP), human gamma globulin (HGG) and control normal serum (CNS) in the absence or presence of chloride-5-methyl-12(H)-chino[3,4-b][1,4]benzothiazine (Salt1) at increasing concentration, a decrease in protein fluorescence was observed. Table 1 shows the percentage of protein fluorescence quenching for the highest concentration of Salt1.

The data collected in Table 1 show that the strongest quenching of protein fluorescence in the presence of ligand with the increase in Salt1 concentration was in the range of 54.34% and 59.00% for AGP at λ_ex_ 275 and λ_ex_ 295 nm, respectively. The ligand’s ability to quench protein fluorescence is related to the energy transfer between the excited fluorophore and ligand chromophore when the distance between them is not more than 10 nm [19]. It means that the quencher (Salt1) is sufficiently close to protein tryptophanyl or/and tyrosyl residues. Due to this phenomenon, it can be assumed that Salt1 has a strong affinity for AGP molecules and weaker interactions with HSA and HGG molecules. The decrease in the intensity of proteins’ emission fluorescence spectra as a result of the interaction with Salt1 at increasing concentration indicates changes in their tertiary structures. This effect is observed also probably due to the exposure of tryptophanyl and tyrosyl residues to the solvent [20]. In the presence of Salt1, slight longwave shifts (red shift) of HGG and CNS (at 3·10^−6^ mol·L^−1^ concentrations) fluorescence bands were observed, and the spectral parameter A was calculated. Spectral parameter A is the ratio of fluorescence intensity at wavelengths λ 365 nm and λ 320 nm ($A=\frac{F_{365\;nm}}{F_{320\;nm}}$), and it has been used because of its sensitivity to small changes in the position of protein maximum fluorescence wavelength (λ_max_) [21]. Moreover, the changes in protein conformation under the influence of Salt1 based on full width at half maximum (FWHM) values have been studied. Table 2 presents changes of wavelength at protein maximum fluorescence(Δλ), parameter A (Δ**A**) and full width at half maximum (ΔFWHM) for the protein in the presence of Salt1 relative to the protein without Salt1.

Similar to the present study, Maciążek–Jurczyk et al. [22] used spectral parameter A and FWHM values to analyze the changes in the tertiary structure of native and oxidized human serum albumin. They observed a decrease in the values of spectral parameter A and FWHM, pointing to the increase in the environmental hydrophobicity of tyrosyl and tryptophanyl residues. Salt1 is a factor that could modify the tertiary structure of a protein, and an observed increase in the values of spectral parameter A and FWHM for HSA, AGP, HGG and CNS in the presence of Salt1 means an increase in the hydrophilicity of tyrosyl and tryptophanyl residues’ surroundings and their exposure to the environment. The occurrence of red shift also indicates changes in the spatial conformation of HSA, AGP, HGG and proteins contained in the control normal serum (CNS) due to the presence of Salt1. Conformational variations in the tertiary structure of protein under the influence of Salt1 have also been manifested by the changes in FWHM values. These phenomena confirm the existence of interaction between the studied molecules and the ligand, as well as the interaction of the ligand with normal control serum. Studies conducted by Maciążek-Jurczyk et al. [7] on changes in the tertiary structure of fibrillated/aggregated and unmodified HSA also proved that spectral parameter A and FWHM values allow the determination of the changes in protein structure.

**Figure 1:**
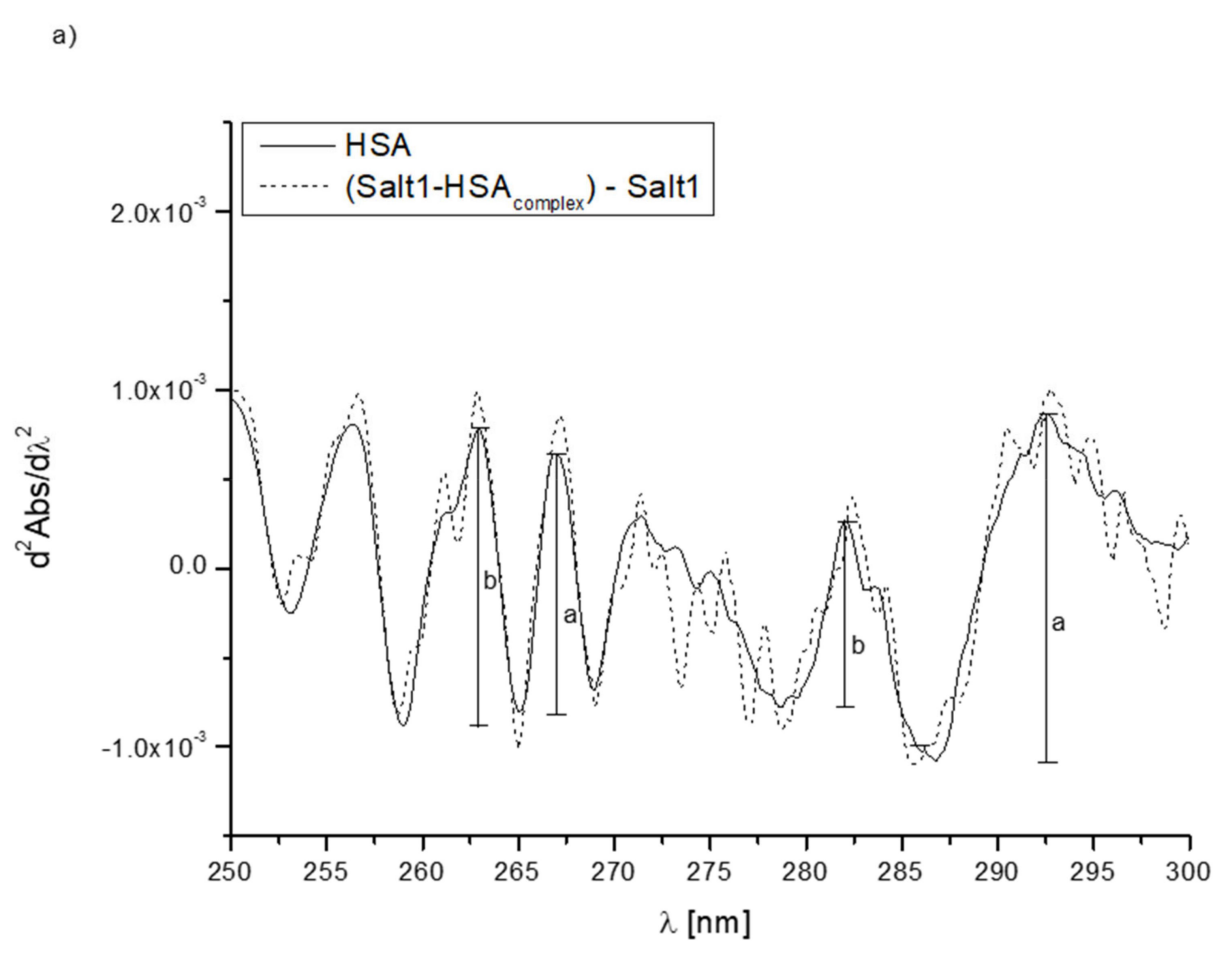

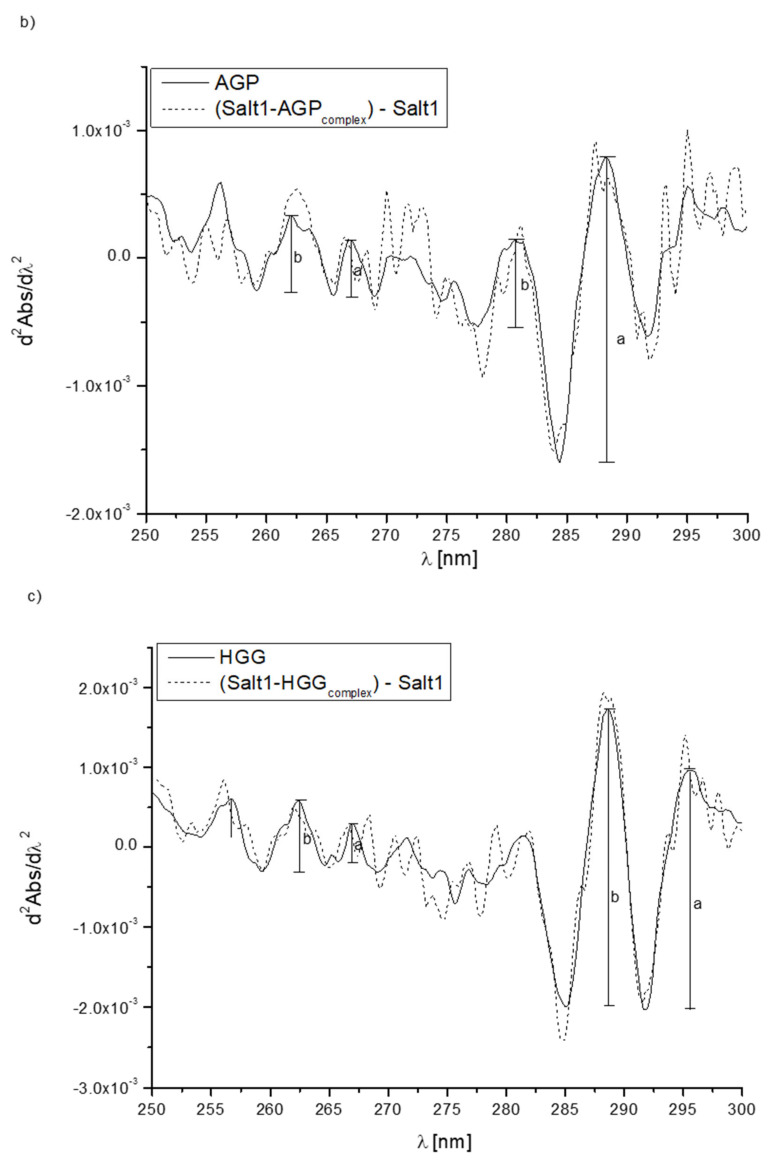

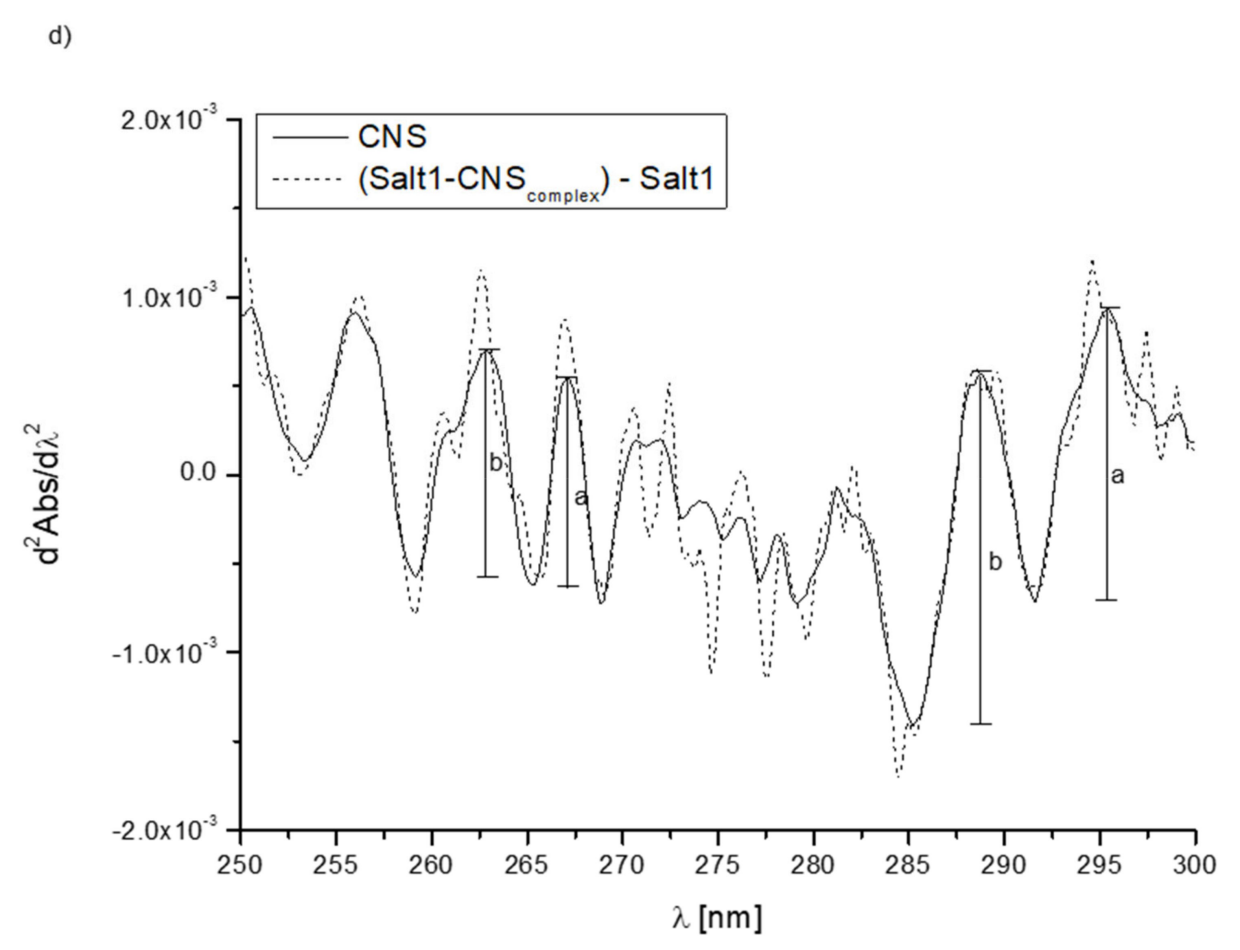
Second derivative absorption spectra of (**a**) HSA (—), (**b**) AGP (—), (**c**) HGG (—) and (**d**) CNS (—) and second derivative differential absorption spectrum of Salt1–protein complex vs. Salt1 (- - - ).

In order to further confirm the effect of Salt1 on the proteins’ tertiary structures in the environment of phenylalanyl, tyrosyl and tryptophanyl residues, second derivatives of differential absorption spectra were recorded. The second derivative of a differential absorption spectrum is a useful parameter in the assessment of changes occurring in the environment of aromatic amino acids, especially if they are in the system in different amounts [23,47]. The appropriate use of second derivative spectroscopy allows the interference caused by the presence of the solvent to be reduced or eliminated. The obtained second derivative of the differential absorption spectrum in the wavelength range from 250 to 270 nm shows changes in the environment within phenylalanyl residues, while in the range between 270 and 290 nm, it shows changes within tyrosyl and tryptophanyl residues [23]. Figure 1 shows the changes in the intensity of the second derivative of differential absorption spectra of HSA, AGP, HGG and CNS due to the presence of Salt1.

Second derivative spectroscopy allows for better detecting slight spectrum features such as shoulders, ripples and their improvement qualification with very low or insignificant error. Due to these properties, it is possible to accurately determine the changes in the environment around the aromatic amino acid residues included in the protein [48]. By the analysis of the obtained spectra, changes due to the presence of Salt1 (shoulders transforming into distinct peaks) have been observed, and in order to confirm the alterations in the environment of phenylalanyl, tyrosyl and tryptophanyl residues of HSA, AGP, HGG and CNS, the parameter *r_p_* was calculated. Parameter$\;r_{p}=\frac{a}{b}$, where $a=a_{1}-a_{2}$ and $b=b_{1}-b_{2}\;$are the $\frac{d^{2}Abs}{d\lambda^{2}}$ values of two adjacent peaks in the phenylalanyl (λ 250–270 nm), tyrosyl and tryptophanyl (λ > 270 nm) absorption regions. *r_p_* values are presented in Table 3 [23,29,49].

The analysis of parameter *r_p_* shows the influence of newly synthesized Salt1 on HSA, AGP, HGG and CNS tertiary structure by changing the environment within aromatic amino acid residues, making it more hydrophilic. Similar studies were conducted by Ichikawa et al. [23]. Using the second derivative of differential absorption spectra, they studied how denaturing agents affect the changes in the spectral intensity, thus assessing the influence of the environment and the amount of phenylalanyl residues in serum albumin, insulin, ribonuclease and lysozyme. Based on the obtained data, they proved that the second derivative of differential absorption spectra is a good method for the evaluation of minor environmental changes in chromophores’ surroundings caused by the presence of an additional factor. Similar studies have been conducted by Terada et al. [49]. Using second derivative spectra, they investigated the influence of various factors on tyrosyl and tryptophanyl residues present in ribonuclease (tyrosine-rich protein), lysozyme (tryptophan-rich protein) and bovine serum albumin.

**Figure 2:**
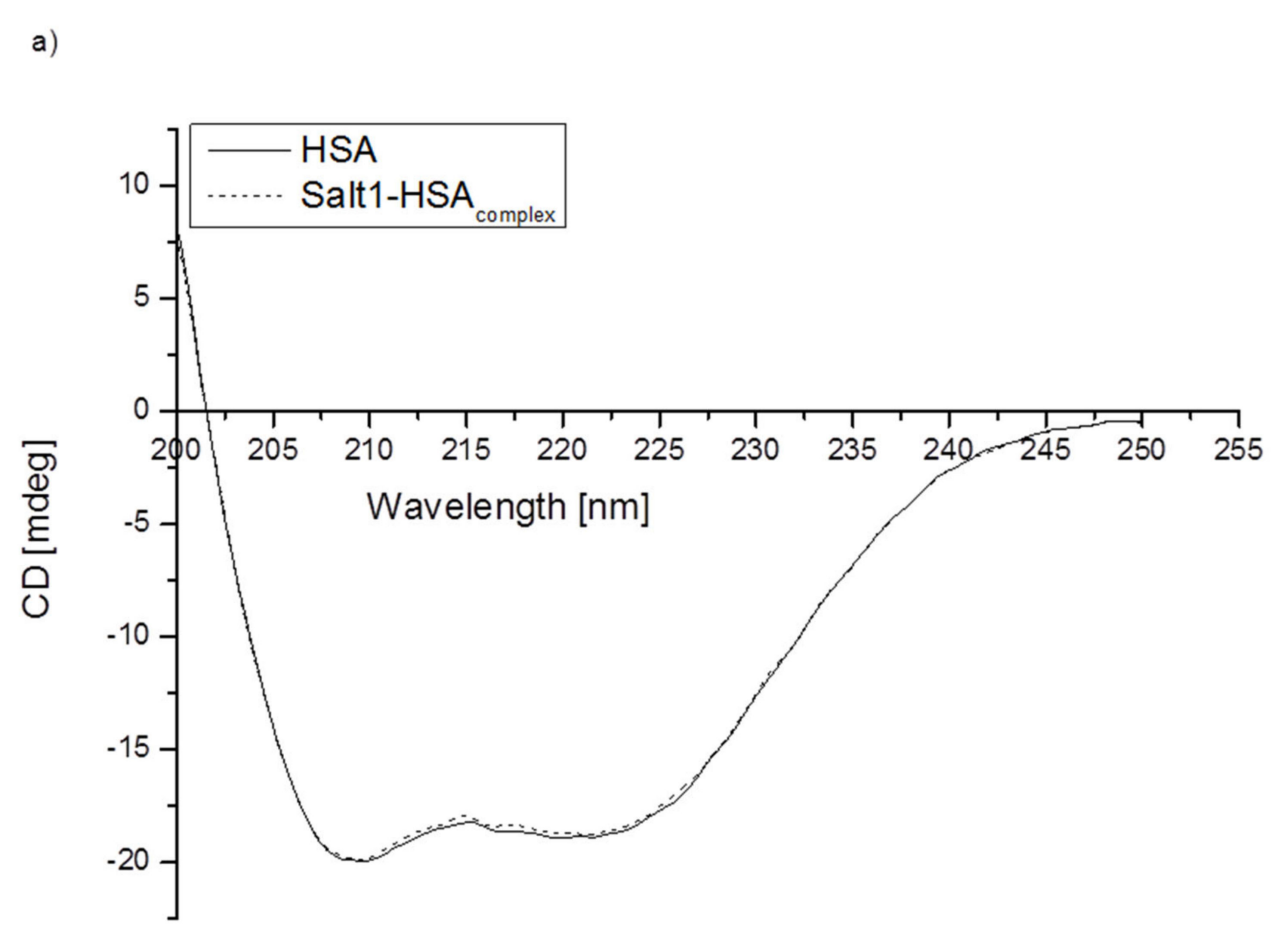

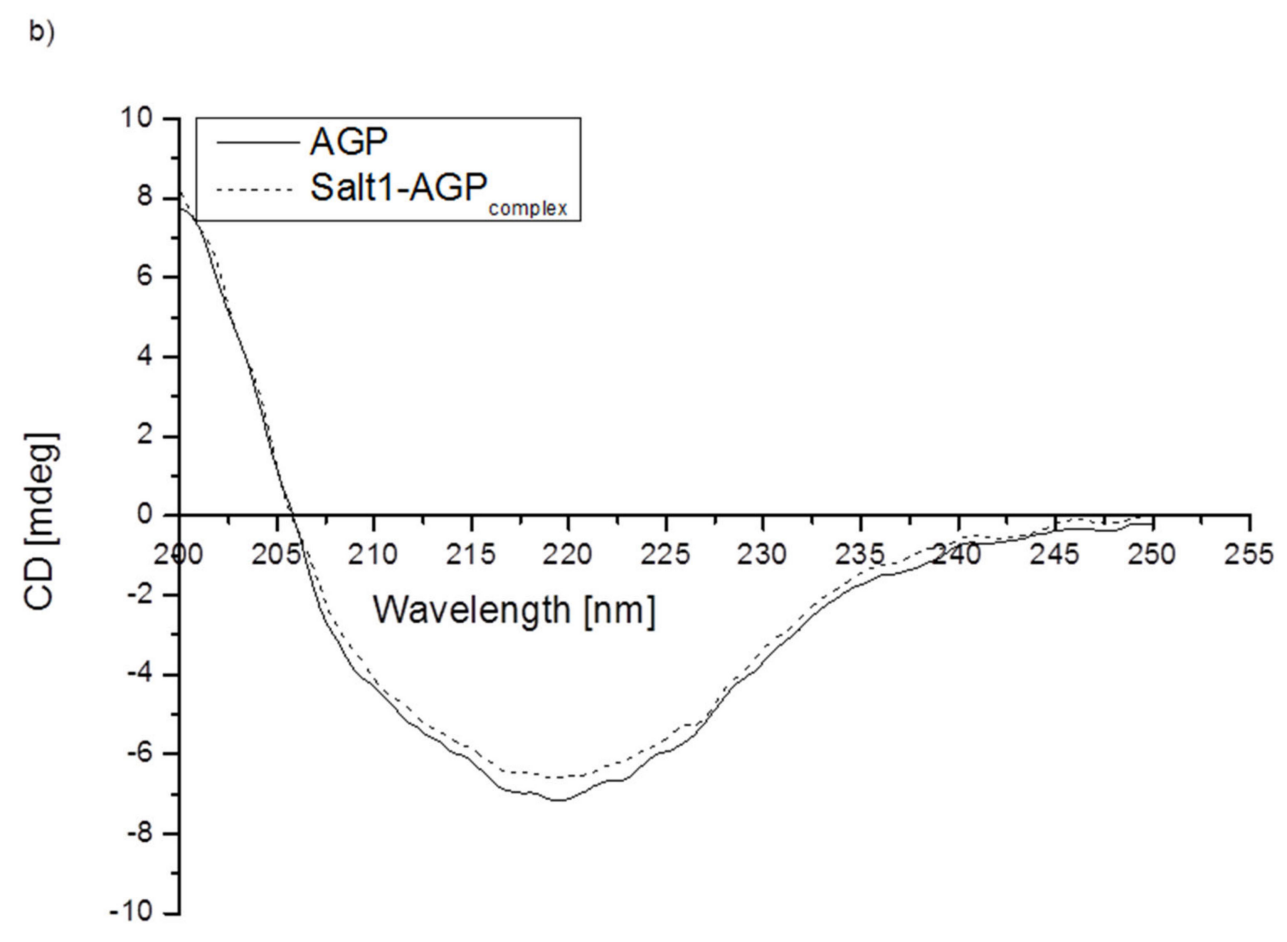
Far-UV circular dichroism spectra of (**a**) HSA and (**b**) AGP in the absence or presence of Salt1.

To assess how the presence of Salt1 influences the secondary structure of proteins, circular dichroism (CD) spectroscopy was used (Figure 2). CD spectroscopy is one of the most successful techniques used for the structural characterization of proteins [50]. It is a practical tool for rapid determination of the secondary structure (far-UV CD) and tertiary structure (near-UV CD). Protein CD is most widely used to determine the folding and binding properties of proteins. Moreover, it can be used to study protein interactions [51]. Due to the fact that HSA and AGP could be the main transporters of Salt1 in the bloodstream, in this study, CD spectroscopy was used to analyze changes in the secondary structure of HSA and AGP by the presence of Salt1. The percentage (%) content of the secondary structure elements of HSA and AGP was obtained using the Secondary Structure Estimation program with Yang’s and Reed’s reference models, respectively, and data are presented in Table 4. Different reference models were used due to the better fit of the curves: unknown and calculated.

As shown in Figure 2, the band intensity of HSA and AGP is not changed significantly by the addition of Salt1. Slight changes in AGP ellipticity in the presence of ligand confirm the strongest interaction with this protein compared to that with HSA (lack of changes in the far-UV CD spectrum of HSA). The increase in the intensity of the obtained Salt1–protein far-UV CD spectra may be due to the influence of external factors [52].

It can be concluded that the binding of Salt1 has a slight influence on AGP and a nonsignificant influence on HSA α-helix and β-sheet contents. From CD analysis, it can be seen that Salt1 bound with human albumin and α-1-acid glycoprotein amino acid residue tertiary structure does not cause the destabilization of protein secondary structure, which could be of clinical importance, due to the fact that disorders of the secondary structure of a protein might lead to various disorders [53].

**Figure 3:**
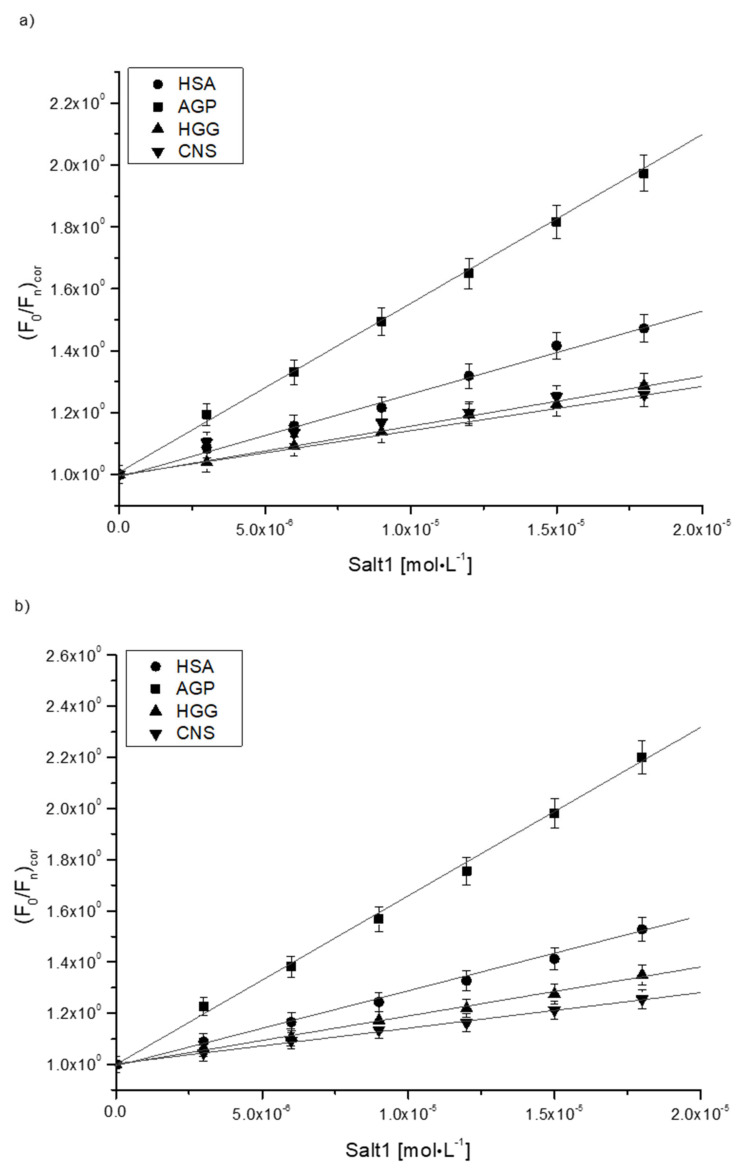
Stern–Volmer plots for the complexes of Salt1 with HSA, AGP, HGG and CNS at (**a**) λ_ex_ 275 nm and (**b**) λ_ex_ 295 nm.

Given that Salt1 interacts with HSA, AGP, HGG and CNS, the Stern–Volmer and Klotz methods were used to characterize the kind of interaction and affinity towards the binding sites. Figure 3a,b presents the Stern–Volmer plots in the complexes of Salt1 with HSA, AGP, HGG and CNS at λ_ex_ 275 nm (Figure 3a) and λ_ex_ 295 nm (Figure 3b).

Based on the Stern–Volmer Equation (3) for the complexes of Salt1 with HSA, AGP, HGG and CNS, the Stern–Volmer constants (K_S-V_) [M^−1^] and bimolecular quenching rate constants (k_q_) were determined and are presented in Table 5.

The K_S-V_ constant is a mean value of the quenching constants characterizing all protein binding sites. It allows us to estimate the protein’s fluorophore availability for Salt1. Unfortunately, it does not give essential information about the alterations in the complex. The K_S-V_ constants obtained based on the Stern–Volmer curve equation (Equation (3)) (Figure 3) describe the distance between the ligand and the excited fluorophores. With the increase in K_S-V_ constant, the distance between the ligand and protein decreases, and the complexes of Salt1 with HSA, AGP, HGG and CNS become stronger. However, a stronger complex means a weaker therapeutic effect [20]. From the data collected in Table 5, it can be observed that the K_S-V_ values are the highest for Salt1–AGP complex (K_S-V_ equals 5.40 ± 0.10·10^4^ and 6.72 ± 0.10·10^4^ mol·L^−1^ at λ_ex_ 275 and 295 nm, respectively). This indicates that Salt1 has a stronger affinity towards the excited fluorophores of α-1-acid glycoprotein than those of other proteins and points to the reduction in the distance between the ligand and macromolecule, probably due to the physicochemical properties of both Salt1 and AGP [44]. Due to the linear Stern–Volmer course (Person’s correlation coefficient equals 0.99), it is not easy to clearly state whether fluorescence quenching is static, dynamic or both; therefore, based on Equation (3) the bimolecular quenching rate constants (k_q_) were calculated (Table 5). k_q_ reflects quenching efficiency or availability of fluorophores to the quencher, and depending on k_q_ values, the quenching type is possible to determine. According to Lakowicz, for dynamic (collisional) fluorescence quenching, the maximum value of k_q_ in aqueous solution is equal to 1·10^10^ mol^−1^·L·s^−1^ [4]. From the data collected in Table 5, it can be concluded that k_q_ values of the order to 10^14^ concern static fluorescence quenching during the formation of Salt1 complexes with HSA, AGP, HGG and CNS. Static quenching taking part in the interaction reduces the intensity of emitted HSA, AGP, HGG and CNS fluorescence at the time when Salt1 in its unexcited state interacting with fluorophore molecules with the decrease in the population of available and excitable fluorophores [54].

The values of K_a_ constants characterize the stability of ligand binding to the protein molecule. In order to determine the association constants (K_a_) for Salt1 complexes with HSA, AGP, HGG and CNS and to identify the number of binding site classes for the independent class of drug binding sites in individual proteins, the Klotz curves were plotted, and the calculated K_a_ values are presented in Table 6.

**Figure 4:**
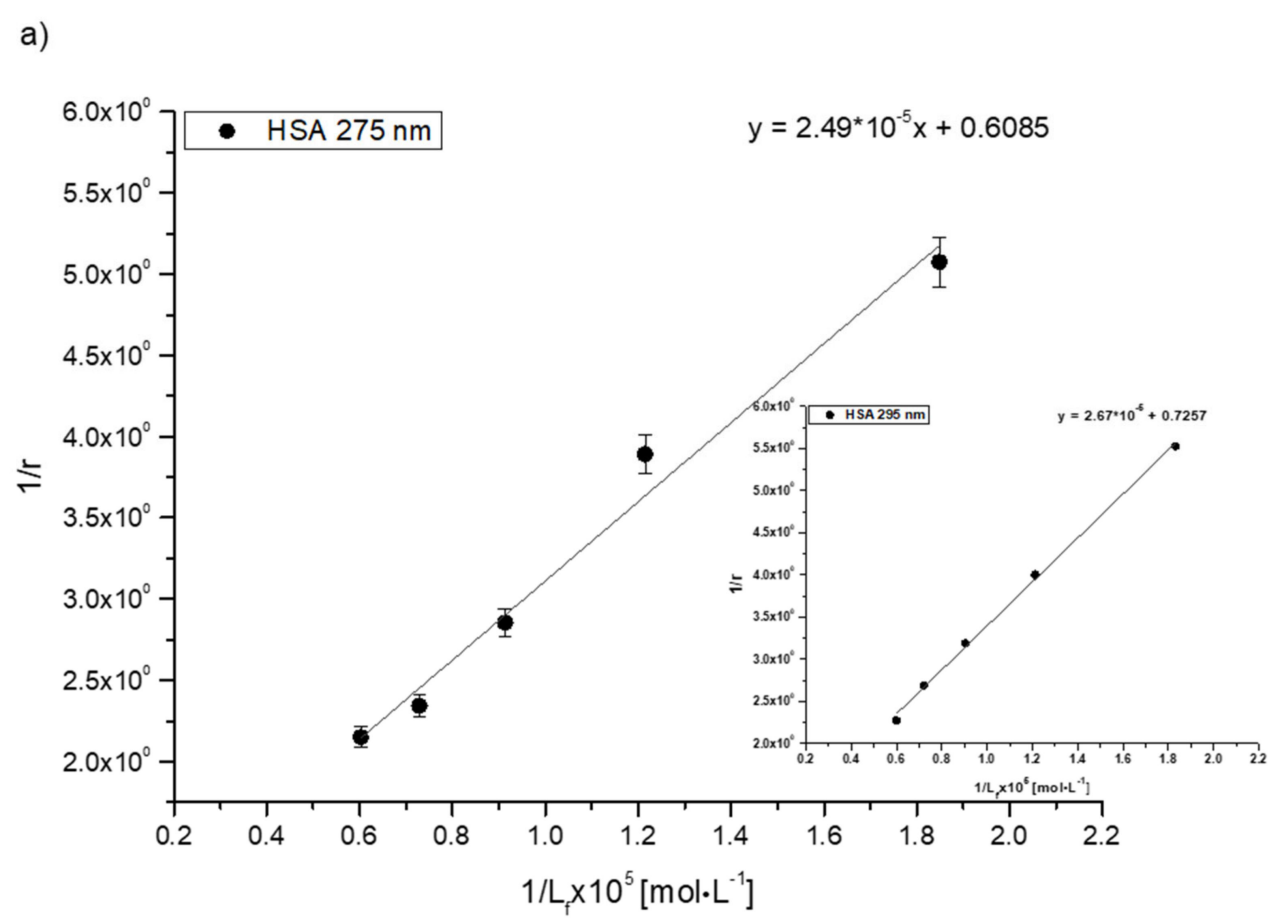

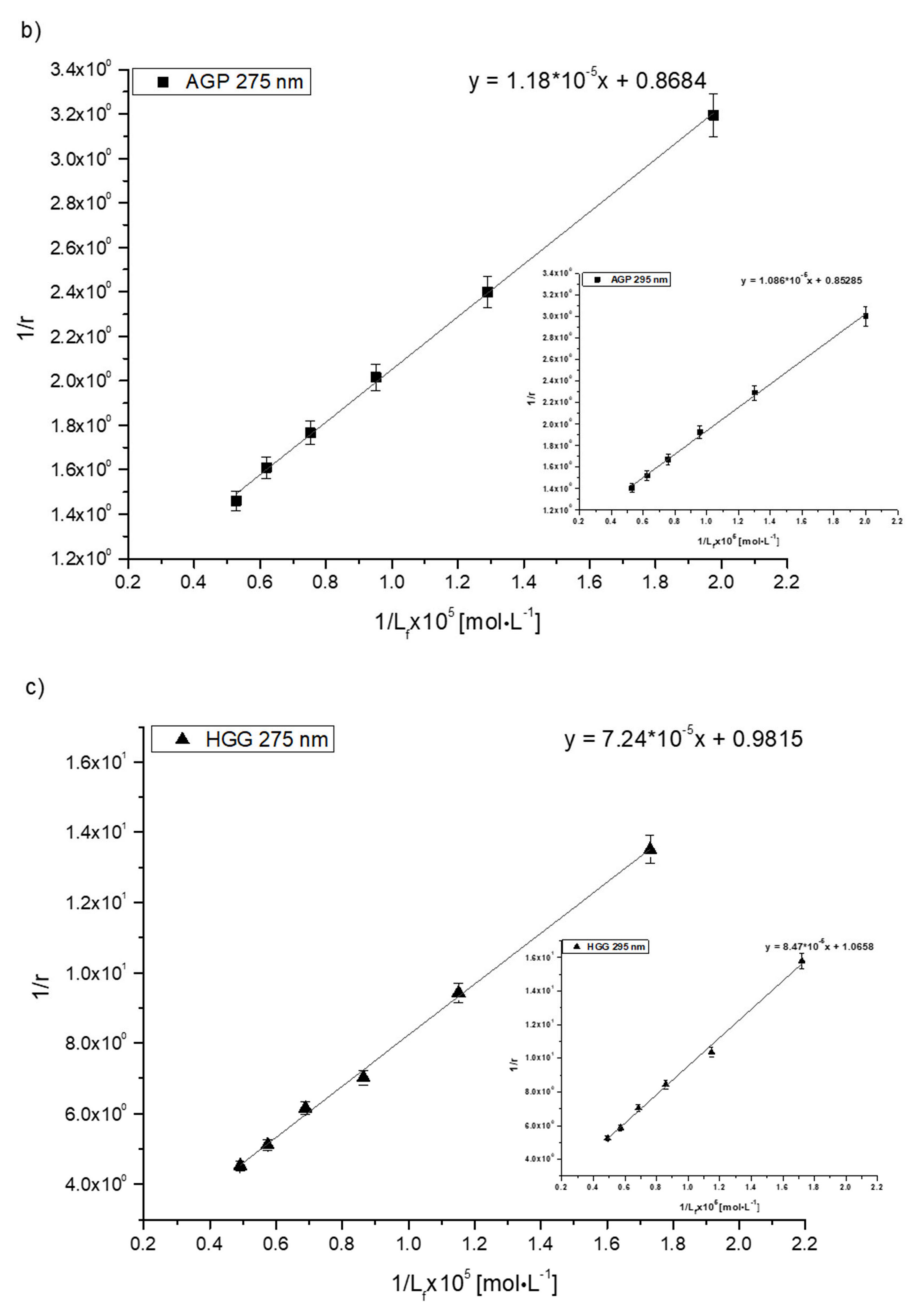

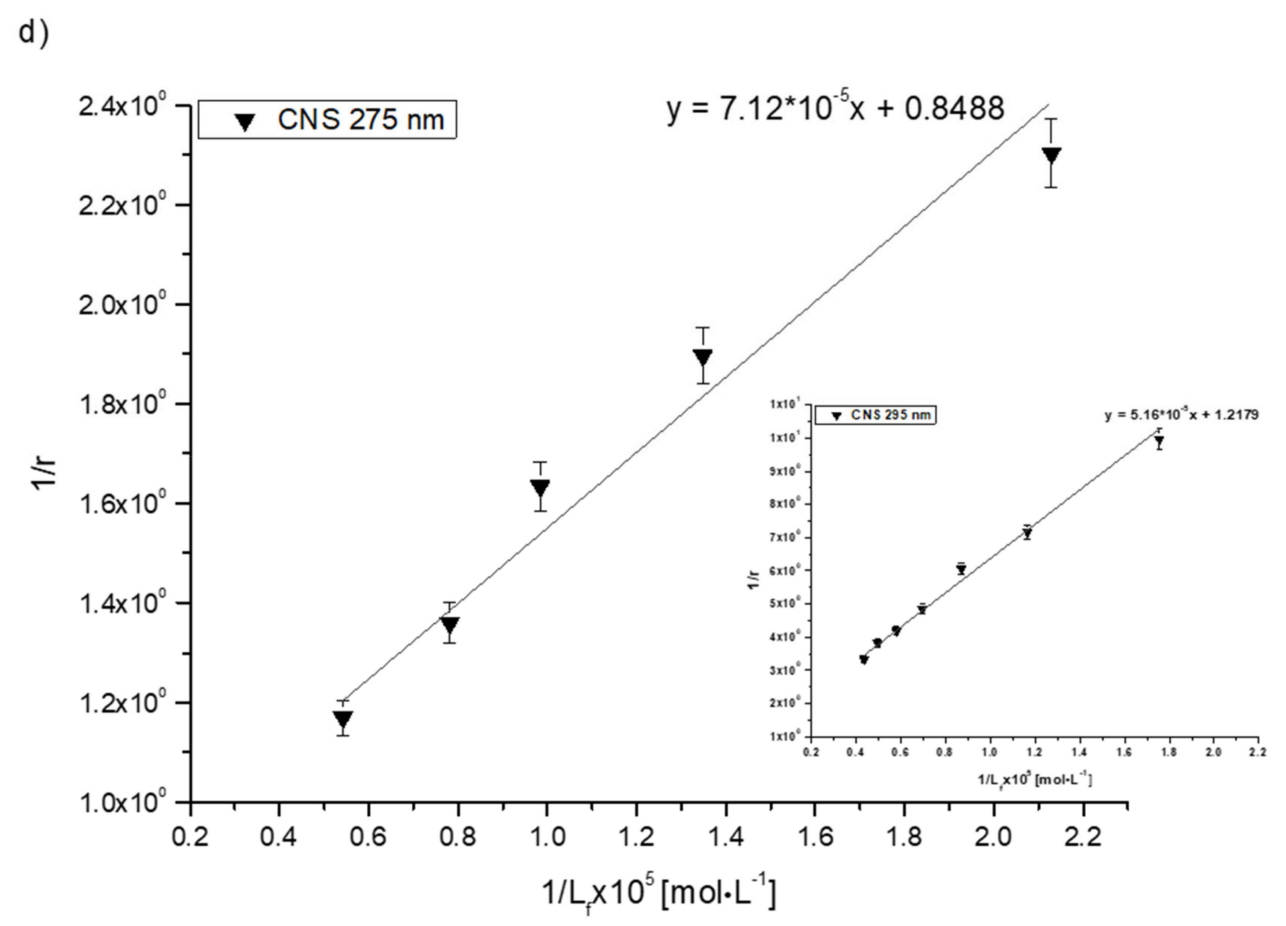
The Klotz plots for (**a**) Salt1–HSA, (**b**) Salt1–AGP, (**c**) Salt1–HGG and (**d**) Salt1–CNS at λ_ex_ 275 nm; inserts showλ_ex_ 295 nm.

Analyzing the values of the association constant K_a_ obtained by the Klotz method (Figure 4, Table 6), it was found that the highest values were calculated for the Salt1–AGP complex: 7.35·10^4^ and 7.86·10^4^ mol·L^−1^ at excitation wavelengths λ_ex_ 275 and 295 nm, respectively. Salt1 forms the strongest complex with α-1-acid glycoprotein, and due to its basic character, AGP is a main binding protein in human plasma [54]. Salt1 forms complexes with HSA and HGG with lower association constant values than with AGP. The formed complexes are weaker, meaning that both HSA and HGG are involved in the distribution of Salt1 in the bloodstream to a lesser extent. This phenomenon may indicate that the release of Salt1 from the complex and achievement of equilibrium is relatively easy to obtain [55]. Complex formation with all selected plasma proteins is favorable to the pharmacological efficacy of the drug due to the variable AGP concentration in plasma depending on the ongoing inflammatory processes [1,2,25,28,29,30,31]. When the concentration of AGP in the blood serum is low, the transport of Salt1 to the target binding site can be completed by HSA and HGG. However, the large K_a_ value for AGP compared to other tested proteins and the high concentration of AGP as a result of inflammation may extend the Salt1 binding effect, which is also a preferred phenomenon in monitoring therapy.

The binding of drugs to plasma proteins is the basis for modulating the efficacy of drug substance concentration at the target site, according to the free drug theory, which assumes that only the free fraction of the drug has a therapeutic effect. After establishing the equilibrium, the concentration of free drug in plasma equals the drug concentration at the target site. Due to the importance of determining the ability of drugs to bind with different plasma proteins, the association constants for the control normal serum (CNS) have been investigated. CNS is a mixture of different human proteins at different known concentrations, established by the manufacturer, imitating the physiological state of the human organism [1]. The values of K_a_ association constants for the Salt1–CNS complex differ depending on the excitation wavelength used. λ_ex_ 275 nm excites tyrosyl and tryptophanyl residues, while λ_ex_ 295 nm excites only tryptophanyl residue [10]. The higher value of K_a_ for the Salt1–CNS complex at λ_ex_ 275 nm excitation wavelength suggests that Salt1 interacts with protein binding sites where both tyrosyl and tryptophanyl residues are located. The control normal serum (CNS) is a mixture of all proteins found in the human body. It can be excited to fluorescence using the same wavelengths, 275 and 295 nm. The obtained values of the association constants confirm the interaction of CNS with Salt1 and/or the formation of the Salt1–CNS complex [1,56,57,58]. Similar values of K_a_ obtained for the CNS and HSA at the excitation wavelength λ_ex_ 295 nm may be due to the fact that HSA is present in the human body in the highest amount and is the main transporting protein [2].

### 2.2. Salt1–Protein Binding Site Assessment

#### 2.2.1. HSA Binding Site Assessment

The concentration of human serum albumin (HSA) is the highest in comparison with other plasma proteins. The higher the HSA concentration, the higher the transport capacity of this protein. This is related to the reversible ability of HSA to bind exogenous and endogenous substances [41]. In the structure of the albumin molecule, two binding sites with known structure and high affinity for drug binding, called Sudlow’s sites, were selected. Sudlow’s site I corresponds to the subdomain IIA, while Sudlow’s site II corresponds to the subdomain IIIA. They are both located in the hydrophobic cavities of the HSA molecule. Sudlow’s sites I and II are completely different in shape, size and drug binding capacity depending on their polarity [47,59,60,61,62,63]. In order to determine the binding sites of Salt1 in the HSA molecule, dansylated amino acids were used. Dansylated amino acids locate in the specific albumin molecule binding sites and are characterized by fluorescent activity. The ability to bind the dansylated amino acid results from its structure. Amino acids that carry an electric charge or have a polar side chain are characteristic of the IIA subdomain, while those that have a hydrophobic side chain in their structure are characteristic of the IIIA subdomain [47,59,60]. Dansyl-L-glutamine (dGlu) and dansyl-L-glycine (dGly) were used to determine the Salt1 binding sites on the HSA molecule. Both of these markers bind to Sudlow’s site I, while dansyl-L-phenylalanine (dPhe) and dansyl-L-proline (dPro) bind to subdomain IIIA [47,59]. Due to the fact that Salt1 is a newly synthesized substance, four markers were used to accurately determine the binding sites of Salt1 with human serum albumin due to the large size of the protein molecule and the complicated binding process [47,59]. Based on the obtained fluorescence emission spectra, the percentage of displacement of dansylated amino acids from the HSA molecule was calculated in relation to the increasing concentration of Salt1 (Equation (1) and Table 7):

(1) $$percentage\;of\;displacement=\frac{F_{0}-F}{F_{0}}\cdot 100\%\;$$

where *F*_0_ and *F* represent fluorescence of the marker in the system with protein and with both protein and ligand, respectively.

From the data collected in Table 7, it can be concluded that with the increase in Salt1 concentration, the percentage of displacement (%) increases. Similar to the study of Ryan et al. [59], this phenomenon means that Salt1 displaces fluorescent markers from their binding sites in the structure of HSA and binds with HSA in both IIA and IIIA subdomains (Table 7).

#### 2.2.2. AGP Binding Site Assessment

α-1-Acid glycoprotein (AGP) is an acidic protein with a positive electric charge. AGP has the shape of a β-barrel surrounded by an α-helix that forms a pocket for the ligands. In the vicinity of the entrances to the ligand-binding pocket, there are two amino acid residues, Arg-68 and Arg-90, having a positive electric charge, which allows interaction with ligands having a negative electric charge [41]. The geometry of the ligand-binding pocket is very complex. The ligand-binding gap is formed by three lobes: the deep lobe I and the large, nonpolar and negatively charged lobes II and III. The third lobe also has a small additional entrance [60]. α-1-Acid glycoprotein (AGP) has a wide and flexible drug binding area that can accommodate two drugs to form a triple complex [61]. Israili and Dayton proved that the AGP molecule contains up to seven binding sites with different properties, but only one of them has significant clinical properties [62]. Quinaldine red (2-[4-(dimethylaminostyryl]-1-ethylquinolone (QR)) was used to confirm the binding site of Salt1 to the AGP molecule. QR is an alkaline-specific fluorescent marker for orosomucoid. After binding to a protein, it strongly fluoresces, while in the unbound form, the intensity of fluorescence is low [23]. Based on the emission spectra and using Equation (1) the percentage displacement of QR from AGP molecule in the presence of Salt1 at increasing concentration was calculated (Table 8).

Based on the obtained data collected in Table 8, it can be concluded that Salt1 displaces the QR from the structure of α-1-acid glycoprotein. The QR displacement percentages are comparable for 1:0.5 and 1:1 molar ratios: 58.5% and 57.0%, respectively. Based on this we can conclude that the QR binding site coincides with the Salt1 binding site in the AGP structure. QR and other substances such as chlorpromazine, warfarin and progesterone were used by Nishi et al. [63] to determine whether the human α-1-acid glycoprotein (hAGP) binding sites overlap with recombinant human α-1-acid glycoprotein (rhAGP). They proved that binding sites of hAGP and rhAGP overlap and QR displacement from AGP molecule by ligand can be a helpful parameter for the evaluation of binding sites.

## 3. Materials and Methods

### 3.1. Chemicals

Human serum albumin, fraction V, Lot No. 2742726 (HSA), dansyl-L-glutamine, Lot No. R22838 (dGlu), and dansyl-L-phenylalanine, Lot No. 8776KA (dPhe), were purchased from MP Biomedicals, Inc. (Illkirch, France). Human gamma globulin, Lot No. 268129/1 32705352 (HGG), and dansyl-L-proline, Lot No. 429136/1 53105105 (dPro), were obtained from Fluka Chemie AG (Buchs, Switzerland). α-1-Acid glycoprotein, Lot No. 049K7565V (AGP), dansyl-glycine, Lot No. 9143321 (dGly), quinaldine red, Lot No. MKBD6820 (QR), and methanol, Lot No. SHBG8324V, were obtained from Sigma-Aldrich Chemie GmbH, (St. Louis, MO, USA), while control normal serum (CNS), Lot 200054/724, was obtained from Alpha Diagnostic (Warszawa, Poland). Chloride-5-methyl-12(H)-chino[3,4-b][1,4]benzothiazine (Salt1) was synthesized in the Department of Organic Chemistry, Faculty of Pharmaceutical Sciences in Sosnowiec, Medical University of Silesia in Katowice, Poland, according to a described procedure [44].

### 3.2. Methods

#### 3.2.1. Sample Preparation

Human serum albumin (HSA) solutions at 2·10^−6^, 3·10^−6^, 5·10^−6^ and 1·10^−5^ mol·L^−1^ concentrations and α-1-acid glycoprotein (AGP), human gamma globulin (HGG) and control normal serum (CNS) at 2·10^−6^, 3·10^−6^ and 1·10^−5^ mol·L^−1^ concentrations, respectively, were incubated at 298 K in 0.05 mol·L^−1^ phosphate buffer at pH 7.4. A stock solution of chloride-5-methyl-12(H)-chino[3,4-b][1,4]benzothiazine (Salt1) at 3·10^−3^ mol·L^−1^ concentration, dansyl-L-proline (dPro), dansyl-L-glutamine (dGlu), dansyl-glycine (dGly) and dansyl-L-phenylalanine (dPhe) at 2.5·10^−3^ mol·L^−1^concentration and quinaldine red (QR) at 3·10^−3^ mol·L^−1^ concentration was prepared in methanol. Ligand–protein binding measurements were conducted at Salt1:HSA 0:1 to 6:1, Salt1:AGP 0:1 to 7:1, Salt1:HGG 0:1 to 7:1 and Salt1:CNS 0:1 to 8:1 molar ratios. For the binding site assessments, HSA and AGP solutions in the absence or presence of fluorescent probes at HSA:dPro 1:1, HSA:dGlu 1:1, HSA:dPhe 1:1 and HSA:dGly 1:1 molar ratios were titrated by Salt1 at 3·10^−6^ to 3.3·10^−5^ mol·L^−1^ concentrations at AGP:QR 1:0.5 and 1:1 molar ratios, they were titrated by Salt1 at 3·10^−6^ to 4.8·10^−5^ mol·L^−1^ concentrations. To determine changes in protein structure, Salt1:protein 4:1 molar ratio was used.

#### 3.2.2. Emission and Absorption Spectra Measurements

The analysis of protein fluorescence quenching by increasing ligand concentration allows the monitoring of intermolecular interactions. A major requirement for fluorescence quenching is the appropriate distance between the excited fluorophore and the ligand [7,8,9,10,11,12,13,14,15,16,17,18,19,20,21,22,23].

The tyrosyl and tryptophanyl residues play a key role in binding studies using fluorescence quenching. To determine binding sites of Salt1 with chosen proteins and control normal serum (CNS), excitation wavelengths λ_ex_ 275 nm (fluorescence emission of tyrosyl and tryptophanyl residues) and λ_ex_ 295 nm (fluorescence emission of tryptophanyl residue) were used.

The fluorescence measurements were recorded at 298 K using fluorescence spectrophotometer JASCO FP-6500 (JASCO International CO., LTD. 4-21, Sennin-cho 2-chome, Hachioji, Tokyo 193-0835, Japan) with quartz cells at 10 mm path length. The accuracy of wavelength was ±1.5 nm. Emission fluorescence spectra of proteins in the presence of Salt1 (Salt1–HSA, Salt1–AGP, Salt1–HGG, Salt1–CNS) were recorded using λ_ex_ 275 nm and λ_ex_ 295 nm, while emission spectra of fluorescent probes in the absence of Salt1 (HSA-dPro, HSA-dGlu, HSA-dPhe, HSA-dGly) and titrated by Salt1 were obtained using λ_ex_ 350 nm. For AGP–QR complexes, in the absence or presence of Salt1, λ_ex_ 500 nm was used. The scattering spectrum of solvent (phosphate buffer) was subtracted from all the spectra.

Due to the absorption of light at both excitation and emission wavelengths (inner filter effect (IFE)), a correction of Salt1–protein system fluorescence intensity was required. Using a JASCO V-530 spectrophotometer (JASCO International CO., LTD. 4-21, Sennin-cho 2-chome, Hachioji, Tokyo 193-0835, Japan), the absorbance measurements at the wavelength used to excite fluorophores and at the emission wavelength, as well as the absorbance measurements for the second derivative of differential spectra in the range between 250 and 300 nm, were made. For the inner filter correction, Equation (2) was used [24]. This equation can be used as long as the absorbance increase of the system is not greater than ~0.3:

(2) $$F_{cor}=F_{obs}\cdot e^{\frac{Aex+Aem}{2}}\;$$

where *F_cor_* and *F_obs_* are the corrected and observed fluorescence (after subtraction of the solvent scattering spectrum), respectively, and *A_ex_* and *A_em_* are the absorbances at the excitation and emission wavelengths, respectively.

The fluorescence quenching effect (static and/or dynamic) of HSA, AGP, HGG and CNS, in the absence or presence of Salt1, was analyzed based on the Stern–Volmer equation (Equation (3)) [4]:

(3) $$\frac{F_{0}}{F}=1+k_{q}\tau_{0}\cdot \left[L\right]=1+K_{SV}\cdot \left[L\right]\;$$

where *F* and *F*_0_ are the fluorescence intensities at the maximum wavelength of albumin in the presence and absence of a quencher, respectively; $k_{q}=\frac{K_{SV}}{\tau_{0}}$ is the bimolecular quenching rate constant in mol^−1^·L·s^−1^; *τ*_0_ is the average fluorescence lifetime of protein without quencher (τ_0HSA_ = 6.000·10^−9^ s [7], τ_0AGP_ = 2.285·10^−9^ s [8], τ_0HGG_ = τ_0CNS_ = 1.000·10^−8^ s); [*L*] is ligand concentration in mol·L^−1^ ([*L*] = [*L_b_*] + [*L_f_*], where [*L_b_*] and [*L_f_*] are the bound and unbound (free) drug concentrations, respectively); and K_S-V_ is the Stern–Volmer constant in mol^−1^·L.

The association constant (*K_a_*) in ligand–protein systems was determined by the Klotz equation (Equation (4)) [9]:

(4) $$\frac{1}{r}=\frac{1}{n}+\frac{1}{n\cdot K_{a}\cdot \left[L_{f}\right]}$$

where r is the number of ligand moles bound to 1 mole of protein,$\;r=\frac{L_{b}}{\left[P\right]}$; *n* is the number of binding site classes; *K_a_* is the association constant in mol^−1^·L; and [*L_f_*] is the free ligand concentration in mol·L^−1^.

#### 3.2.3. Circular Dichroism (CD) Measurements

Far-UV CD spectra of HSA and AGP were recorded using a JASCO J-1500 CD spectropolarimeter equipped with a thermostatic Peltier cell holder (JASCO International CO., LTD. 4-21, Sennin-cho 2-chome, Hachioji, Tokyo 193-0835, Japan) with an accuracy of ± 0.05 °C. Circular dichroism measurements were made in a nitrogen atmosphere at 25 °C in a 1 mm path length quartz cuvette. Samples were scanned from 200 to 250 nm at wavelength intervals of 0.2 nm. Prior to the calculation of the final ellipticity, CD protein spectra were corrected by subtraction of spectra obtained for phosphate buffer, pH 7.4 ± 0.1, measured under identical conditions. Then, using the Savitzky and Golay filters method and a convolution width of 13, the obtained spectra were smoothed. CD intensity is expressed as mean residue ellipticity at wavelength λ (*[θ]_mre_*) according to Equation (5) [10]:

(5) $${\left[\theta \right]}_{mre}=\frac{MRW\cdot \theta_{\lambda }}{10\cdot l\cdot c}\;\left[deg\cdot cm^{2}\cdot dmol^{-1}\right]$$

where *MRW* is mean residue weight (*MRW*_HSA_ = 113.7 Da, *MRW*_AGP_ = 236.3 Da), *θ_λ_* is observed ellipticity at wavelength *λ* in degrees, l is optical path length in cm and c is protein concentration in g·cm^−3^.

### 3.3. Statistics

The results of the study were expressed as a mean ± relative standard deviation (SD) from three independent experiments. Linear regression was analyzed using OriginPro version 8.5 SR1 software (Northampton, MA, USA) by fitting experimental data to the corresponding equation.

## 4. Conclusions

The main goal of the study was to analyze a quinobenzothiazine derivative (Salt1) with anticancer potential in terms of the interaction with human serum proteins (HSA, AGP, HGG) and control normal serum (CNS) using spectrofluorescence, UV-Vis and circular dichroism (CD) spectroscopy. During Salt1–protein complex formation, the environment of amino acid residues taking part in the interaction becomes less hydrophobic and more polar. The qualitative analysis revealed that the most strongly binding protein for Salt1 is probably α-1-acid glycoprotein (AGP). It is noteworthy that both human serum albumin (HSA) and human gamma globulin (HGG) take part in Salt1 distribution in the bloodstream. Using control normal serum (CNS), which is a mixture of all transport proteins found in the human bloodstream, ligand–protein interactions were confirmed. No studies have been registered concerning the in vitro spectroscopic analysis of quinobenzothiazine derivatives in terms of the interaction with human plasma proteins. Despite the fact that the conducted studies are basic, from the scientific point of view, they are novel and encourage further in vitro and in vivo investigations.

## Figures and Tables

**Table 1:** Protein fluorescence quenching at λ_ex_ 275nm and λ_ex_ 295 nm.

Salt1–Protein Complex	λ_ex_ 275 nm	λ_ex_ 295 nm
	Percentage of Fluorescence Quenching (%)	
**Salt1–HSA_complex_**	32.04	34.51
**Salt1–AGP_complex_**	54.34	59.00
**Salt1–HGG_complex_**	25.20	28.99
**Salt1–CNS_complex_**	25.47	25.43

**Table 2:** Concentrations (mol·L^−1^) at the excitation wavelengths λ_ex_ 275 nm and λ_ex_ 295 nm.

Protein	λ_ex_ 275 nm			λ_ex_ 295 nm		
	Δλ_max_ (nm)	ΔA	ΔFWHM (nm)	Δλ(nm)	ΔA	ΔFWHM (nm)
**HSA**	6	0.09	0.37	5	0.14	1.41
**AGP**	2	0.08	2.42	6	0.13	3.62
**HGG**	2	0.06	1.59	6	0.07	5.17
**CNS**	2	0.08	1.87	3	0.11	4.26

**Table 3:** *r_p_* values calculated from the second derivative differential absorption spectra of phenylalanyl and tyrosyl/tryptophanyl residues of HSA, AGP, HGG and CNS in the presence of Salt1 at Salt1:protein 4:1 molar ratio.

	**λ 250–270 nm**	**λ > 270 nm**	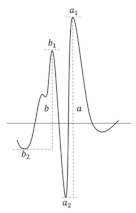
	**$r_{p}=\frac{a\;}{b}$**	**$r_{p}=\frac{a}{b}$**	
**HSA**	0.87	1.86	
**Salt1–HSA_complex_**	1.03	1.97	
**AGP**	1.19	3.93	
**Salt1–AGP_complex_**	0.67	4.10	
**HGG**	0.54	0.80	
**Salt1–HGG_complex_**	1.32	0.34	
**CNS**	0.91	0.84	
**Salt1–CNS_complex_**	1.36	0.89	

**Table 4:** The percentage (%) content of the secondary structure elements of HSA and AGP based on Yang’s and Reed’s reference models, respectively.

	[θ]_mrw_ at 209.8 nm (mdeg·cm^2^·dmol^−1^)	[θ]_mrw_ at 219.4 nm (mdeg·cm^2^·dmol^−1^)	% α-Helix	% β-Sheet	% Turn	% Random
**HAS ^a^**	−17063.3333	−16147.26496	37.7	10.8	21.0	30.4
**Salt1–HSA_complex_^a^**	−16991.3675	−16028.88890	37.3	10.1	21.6	30.0
**AGP ^b^**	-	−7892.39560	17.1	82.9	-	-
**Salt1–AGP_complex_^b^**	-	−7257.86810	14.9	85.1	-	-

^a^ Yang’s reference model, ^b^ Reed’s reference model.

**Table 5:** The Stern–Volmer (K_S-V_) and bimolecular quenching rate constants (k_q_) for Salt1 in complexes with HSA, AGP, HGG and CNS.

	λ_ex_ 275 nm			λ_ex_ 295 nm		
	Pearson’s	$(K_{s-v}\cdot$ 10^4^) ±SD * (mol·L^−1^)	$(k_{q}\cdot$ 10^14^) ±SD * (mol^−1^·L·s^−1^)	Pearson’s	$(K_{s-v}\cdot$ 10^4^) ±SD * (mol·L^−1^)	$(k_{q}\cdot$ 10^14^) ±SD * (mol^−1^·L·s^−1^)
**Salt1–HSA_complex_**	0.997	2.70 ± 0.10	0.43 ± 0.10	0.998	2.84 ± 0.08	0.46 ± 0.08
**Salt1–AGP_complex_**	0.999	5.40 ± 0.10	2.40 ± 0.10	0.998	6.72 ± 0.20	2.94 ± 0.02
**Salt1–HGG_complex_**	0.998	1.60 ± 0.03	0.81 ± 0.03	0.996	1.14 ± 0.04	0.11 ± 0.04
**Salt1–CNS_complex_**	0.990	1.28 ± 0.10	0.14 ± 0.10	0.999	1.38 ± 0.02	0.14 ± 0.02

* SD—standard deviation.

**Table 6:** The binding parameters for Salt1–HSA, Salt1–AGP, Salt1–HGG and Salt1–CNS systems.

	λ_ex_ 275 nm		λ_ex_ 295 nm	
	$(K_{a}\cdot$ 10^4^) ±SD * (mol·L^−1^)	*n* ± SD *	$(K_{a}\cdot$ 10^4^) ±SD * (mol·L^−1^)	*n* ± SD *
**Salt1–HSA_complex_**	2.45 ± 0.01	1.62 ± 0.10	2.71 ± 0.01	1.38 ± 0.10
**Salt1–AGP_complex_**	7.35 ± 0.01	1.15 ± 0.02	7.86 ± 0.03	1.17 ± 0.01
**Salt1–HGG_complex_**	1.41 ± 0.02	1.01 ± 0.20	1.33 ± 0.02	0.94 ± 0.20
**Salt1–CNS_complex_**	6.35 ± 0.01	0.95 ± 0.10	2.36 ± 0.02	0.82 ± 0.20

* SD—standard deviation.

**Table 7:** The percentage of displacement of dansylated amino acids from the HSA molecule in the presence of Salt1 concentration; [HSA] = [dGlu] = [dGly] = [dPhe] = [dPro] = 5·10^−6^ mol·L^−1^; λ_ex_ 350 nm.

C_Salt1_ (mol·L^−1^)	[HSA]:[dGlu]	[HSA]:[dGly]	[HSA]:[dPhe]	[HSA]:[dPro]
	1:1			
	percentage of displacement (%)			
**0**	-	-	-	-
**3·10^−6^**	7.43	5.19	2.99	7.53
**6·10^6^**	12.35	9.68	5.75	11.75
**9·10^−6^**	15.85	13.59	8.98	15.53
**12·10^−6^**	20.72	18.80	11.78	20.98
**15·10^−6^**	24.13	22.87	14.84	24.11
**18·10^−6^**	27.78	25.68	18.30	29.09
**21·10^−6^**	31.27	29.79	21.29	31.61
**24·10^−6^**	34.20	32.80	23.88	36.83
**27·10^−6^**	36.34	35.61	25.81	39.78
**30·10^−6^**	38.75	37.76	27.79	41.66
**33·10^−6^**	40.99	41.35	30.31	44.82

**Table 8:** The percentage of displacement of QR from the AGP molecule in the presence of Salt1 concentration; [AGP] = [QR] = 3·10^−6^ mol·L^−1^; λ_ex_ 500 nm.

C_Salt1_ (mol·L^−1^)	[AGP]:[QR] 1:0.5	[AGP]:[QR] 1:1
	percentage of displacement (%)	
**0**	-	-
**3·10^−6^**	9.2	10.7
**6·10^−6^**	14.8	18.9
**12·10^−6^**	26.8	26.4
**18·10^−6^**	32.7	34.8
**24·10^−6^**	41.2	39.8
**30·10^−6^**	46.2	46.1
**36·10^−6^**	49.3	50.3
**42·10^−6^**	51.8	52.6
**48·10^−6^**	58.5	57.0

## Data Availability

Not applicable.
